# Human-Centered Design and Iterative Refinement of Tools and Methods to Implement a Surveillance and Risk Prediction System for Clinical Deterioration in Ambulatory Cancer Care

**DOI:** 10.1055/a-2437-9977

**Published:** 2025-02-21

**Authors:** Daniel J. France, Paromita Nath, Jason Slagle, Shilo Anders, Megan Salwei, Timothy Vogus, Hannah Slater, Carrie Reale, Laurie Novak, Lori-Anne Parker-Danley, Zachary Kohutek, Rajiv Agarwal, Joyce Harris, Barbara Yudiskas, Ralph Conwill, Terrell Smith, Evan Rhodes, Emma Schremp, Erin A. Gillaspie, Adam Wright, Robert E. Freundlich, Ryan Myles, Janelle Faiman, Sankaran Mahadevan, Matthew Weinger

**Affiliations:** 1 Department of Anesthesiology, Vanderbilt University Medical Center, Nashville, Tennessee, United States; 2 Vanderbilt University School of Nursing, Nashville, Tennessee, United States; 3 Department of Mechanical Engineering, Henry M. Rowan College of Engineering, Glassboro, New Jersey, United States; 4 Department of Biomedical Informatics, Vanderbilt University Medical Center, Nashville, Tennessee, United States; 5 Owen Graduate School of Management, Vanderbilt University, Nashville, Tennessee, United States; 6 Vanderbilt University Medical Center, Nashville, Tennessee, United States; 7 Department of Patient and Family Engagement, Vanderbilt University Medical Center, Nashville, Tennessee, United States; 8 Department of Radiation, Vanderbilt University Medical Center, Nashville, Tennessee, United States; 9 Department of Medicine, Vanderbilt University Medical Center, Nashville, Tennessee, United States; 10 Department of Patient Care, Vanderbilt University Medical Center, Nashville, Tennessee, United States; 11 Department of Pediatrics, Vanderbilt University Medical Center, Nashville, Tennessee, United States; 12 Department of Thoracic Surgery, Vanderbilt University Medical Center, Nashville, Tennessee, United States; 13 Department of Civil Engineering, Vanderbilt University, Nashville, Tennessee, United States

**Keywords:** surveillance-and-risk prediction, ambulatory cancer, wearables, patient-reported outcome measures, non-routine events, patient-centered care

## Abstract

**Background** A common cause of preventable harm is the failure to detect and appropriately respond to clinical deterioration. Timely intervention is needed, particularly in medically complex patients, to mitigate the effects of adverse events, disease progression, and medical error. This challenging problem requires clinical surveillance, early recognition, timely notification of the appropriate clinicians, and effective intervention.

**Objectives** We determined the feasibility of designing, developing, and implementing the tools and processes to create a surveillance-and-risk prediction system to detect clinical deterioration in cancer outpatients.

**Methods** We used systems engineering and iterative human-centered design to develop a functional prototype of a surveillance-and-risk prediction system. The system includes passive surveillance involving wearable sensors, active surveillance involving patient event and symptom reporting as well as extraction of selected patient data from the electronic health record (EHR), a predictive model, and communication of estimated risk to clinicians. System usability was evaluated using patient and clinician interviews and clinician ratings using the System Usability Scale (SUS).

**Results** Fifty of 71 recruited patients enrolled in the feasibility study. Patient-reported outcome measures and clinical data extracted from the EHR were the best predictors of a patient's 7-day risk of experiencing unplanned treatment events (UTEs, i.e., emergency room visits, hospital admissions, or major treatment changes). Deep learning neural network models using these predictors demonstrated modest performance in predicting 7-day UTE risk (PROMS, F-measure: 0.900, area under the receiver operating characteristic curve [AUC-ROC]: 0.983; clinical data from EHR F-measure: 0.625, AUC-ROC: 0.983). Patient risk scores were communicated to clinicians using a risk communication prototype rated favorably by clinicians with a SUS score of 76 out of 100 (median = 80; range: 60–85).

**Conclusion** We demonstrate the feasibility of a surveillance-and-risk prediction system for detecting and reporting clinical deterioration in cancer outpatients. Future research is needed to fully implement and evaluate system adoption and effectiveness under different clinical situations.

## Background and Significance

More than 1.6 million new cases of cancer occur annually in the United States. 1 Cancer often requires multimodal therapy coordinated by multiple providers. 2 3 The National Academy of Medicine declared a *crisis in quality cancer care* , calling it, among other things, insufficiently patient-centered. 1 Cancer surgery, chemotherapy, and radiotherapy are all associated with significant treatment toxicity; more than 50% of elderly cancer patients receiving chemotherapy have at least one severe toxicity. 4 Further, cancer care provides multiple opportunities for medical errors and associated harm. 5 6 7 8 9 10 11 12 Oncology patients, especially those who are elderly, have multiple comorbidities, or low socioeconomic status, are at particular risk of unexpected clinical deterioration from treatment toxicities or preventable harm (e.g., unplanned ER visit, hospitalization, or major change in care plan). 13 14

Cancer is increasingly being treated in ambulatory settings where clinicians are not immediately available to intervene. Outpatients recovering from an acute event (e.g., surgery), experiencing illness postdischarge, or those undergoing chemo- or radiotherapy are at high risk for clinical deterioration. Early signs of deterioration can be missed, leading to the need for more acute care (e.g., admission 15 ) and preventable harm. For cancer patients, preventable admissions range from 2 to 19% in academic cancer centers to 31% in community hospitals. 5 6 16

Preventing harm from unexpected clinical deterioration requires timely detection and response. Numerous surveillance systems have been developed for inpatients including continuous monitoring technologies 17 18 and early warning systems. 19 20 21 Such technologies and systems have had limited application for deterioration detection in outpatient cancer care 22 23 24 25 26 where low-tech solutions prevail (e.g., more frequent clinic visits, phone calls 27 28 ). Current approaches still largely rely on patients and/or caregivers to recognize early signs of deterioration and appropriately communicate this to the clinical system.

## Objectives

Herein, we describe a proof-of-concept study involving the human-centered design (HCD) 29 30 and the development of a technology-facilitated clinical deterioration surveillance-and-response system for ambulatory cancer patients. The goals of our study were to (1) gain a thorough understanding of the issues, opportunities, and challenges associated with the reliable detection of, and response to, unexpected deterioration across all stakeholders; (2) design and prototype a surveillance-and-risk prediction system; and (3) evaluate the prototype by engaging clinicians in a realistic vignette-based usability study. In this paper, we describe three phases encompassing prototype development and evaluation.

## Methods

### Study Design

In the first phase, we used systems engineering and HCD to iteratively develop the surveillance system which consisted of both active (e.g., patient-reported outcome measures [PROMS] 27 ) and passive (e.g., data extracted from the electronic health record [EHR]) components. The surveillance system also used wrist activity monitors and smartphones. In the second phase, we used this system to collect surveillance and outcome data including patient reporting of non-routine events (NREs), defined as any deviation from optimal care. 28 31 In the third phase, we developed a risk communication system (RCS) and also conducted a simulated clinical evaluation. Based on phase 2 data, we developed a preliminary predictive model of 7-day risk for experiencing an unplanned treatment event (UTE).

### Setting and Participants

The study occurred in the outpatient cancer clinics within a National Cancer Institute (NCI)-designated Comprehensive Cancer Care Center. Inclusion criteria for patient participants were adults (age ≥18) with a diagnosis of any stage of head or neck, lung, esophageal, or pancreatic cancer who were receiving curative surgery, chemotherapy, and/or radiotherapy, able to provide informed consent, and willing to participate for at least 3 months. These cancer types were selected based on the complexity of their treatment regimens and relatively high incidence of adverse events compared with other cancer types. A heterogenous sample of cancer types was also selected to determine the common and unique design requirements for the prototype system across cancer clinics. Family caregivers, who supported the care of eligible cancer patients, as well as physicians and staff involved in treating these patients were encouraged to co-participate in the surveillance system.

### Unexpected Clinical Deterioration

The primary outcome was the incidence of UTEs, defined as a major unanticipated change in care requirements including unplanned emergency department (ED) visits, hospital admissions, or major change in treatment plan. UTEs could be reported by patients using a custom smartphone application (MyCap™) or directly to researchers or clinicians during clinic visits. UTEs were concurrently identified through weekly EHR queries.

### Non-Routine Events

During the weekly interaction with the study patients, we obtained their or their caregiver's reports of NREs. An NRE is defined as *any event that deviates from expected or optimal care for a specific patient in a specific situation* . For our patients, an NRE was generally a care situation that deviated substantively from the care they expected or desired to receive. For example, a patient has symptoms (for example, blistering) that she was not expecting and has not been able to reach the doctor for several days to discuss. Clinicians could also report NREs although this was uncommon. Clinician co-investigators regularly reviewed the reported NREs to code for various contributory factors and to identify potential UTEs.

### Phase 1: Developing and Deploying a Surveillance System

#### Passive Surveillance

For passive surveillance, patient participants used a low-cost activity monitor (Fitbit Charge 2), a smartphone, either owned by or provided to the patient, and the hospital's EHR. The activity monitor was used to collect the following moment-to-moment health and activity data: calories, sedentary and active minutes, sleep, steps walked, and heart rate (resting, average, minimum, and maximum). Due to the irregularity of patient weight recorded in the EHR, we gave all study patients a Bluetooth-enabled smart scale to collect and transmit at-home weights to the activity monitor. With patient approval, we collected geolocation data from their smartphone's Google Maps app. To measure patient activity outside their homes, we captured up to 10 patient-selected “when I'm healthy” (e.g., church, gym, sibling's house, grocery) and “health care” locations (e.g., preferred pharmacy, hospital, ED). A custom computer algorithm that applied prespecified temporal and spatial rules to the global positioning system (GPS) data determined if patient trips outside the home qualified as visits to the patient-designated “healthy” or to health care locations.

We also extracted from the EHR prespecified clinical variables as potential biomarkers for clinical deterioration in cancer outpatients. Using a modified Delphi methodology, eight oncologists reviewed and prioritized a comprehensive list of 33 EHR-based clinical variables based on how likely that variable could reliably predict incipient clinical deterioration. Specific candidate variables were from the following categories: patient demographics (e.g., race, ethnicity), clinical encounter patterns (number of radiation encounters, number of infusions, number of missed appointments), laboratory results (albumin, protein, bilirubin, etc.), outpatient medications (e.g., chemotherapy, pain, nausea, etc.), substance use (e.g., tobacco, alcohol, illicit drugs), and vital signs (e.g., weight, blood pressure, etc.).

#### Active Surveillance

We used the MyCap™ smartphone app 32 to collect PROMs on their smartphones. The data were sent from MyCap directly to a REDCap™ database. We trained and encouraged patients and family caregivers to use MyCap to report NREs *anytime* they experienced them and to also fill out PROMs on a weekly basis. A phone notification from the MyCap app alerted patients each Tuesday to go into the app and fill out a survey titled “Tell Us About Your Week.” NREs were reported using the previously validated Patient Comprehensive OpeN-Ended Survey (PCONES) which elucidates patient-reported NREs. 33 PROMS included completion of a weekly National Comprehensive Cancer Network (NCCN) distress thermometer, 34 NCCN symptoms list, a Global Health Score, 35 and selected items from the Consumer Assessment of Healthcare Providers and Systems (CAHPS®). The questions addressed patients' experiences of problems related to social determinants of health (e.g., transportation, childcare, housing), family (e.g., children, partner, family health), emotional state (e.g., depression, fears, nervousness), and about the quality of the patient's interactions with their care team (e.g., communication and listening).

As a secondary mode of PROM capture, the clinical team prompted patients and family caregivers about events that they had experienced recently (i.e., since their last clinic visit) at each clinic visit.

### Phase 2: Building a Predictive Model

We developed independent statistical models for each of the four components of our surveillance system—PROMS, EHR data, Fitbit data, and geolocation data—to predict the class probabilities that a patient would experience one or more UTEs within the next 7 days. The models were implemented in Python using the scikit-learn library. Prior to model development, dimension reduction techniques, including Pearson correlation analysis, were applied to the set of candidate predictor variables to eliminate redundant variables from each model. Ensemble learning techniques were applied to the model outputs to calculate a 7-day UTE risk score for each patient. Since UTEs are rare events, they were oversampled using the Synthetic Minority Oversampling Technique (SMOTE) to balance the dataset. 36 A random forest classification model was trained to predict 7-day UTE risk. The random forest model combines multiple decision trees, where each tree is trained on a random segment of the predictor space. The set of splitting rules used to segment the predictor space structurally resembles a tree. The output of the random forest model is determined by combining the predictions of individual trees (e.g., a majority vote for classification or an average value for regression).

We determined the accuracy of the risk prediction model by calculating the mean of stratified five-fold cross-validation with 10 repeats. We used sensitivity, specificity, positive predictive value (PPV), negative predictive value, F-measure, and the area under the receiver operating characteristic curve (AUC-ROC) to evaluate model performance. The final predictive model's performance was determined by calculating the mean of the class probabilities from the random forest models built for each data stream. This ensemble technique aims to enhance the overall robustness and reliability of prediction by fusing information from multiple sources, that is, PROMS, EHR data, Fitbit data, and geolocation data.

### Phase 3: Human-centered Design of the Risk Communication System

#### Understanding Users' Needs and the Use Environment

We conducted 36 observations (over 80 hours and across 100 patient encounters) of clinicians, patients, and family caregivers in the cancer clinics to gain a thorough understanding of ambulatory cancer care and to develop preliminary design guidelines for the surveillance-and-risk prediction system. Observations focused on general clinic operations, including patient flow, information flow, and clinician workflow. We also observed clinical processes related to patient and family caregiver engagement, care team interactions, and clinician–technology interactions.

We also conducted 17 interviews with oncology clinicians to understand the processes by which clinical deterioration is detected, communicated, and managed (see Supplementary Material S1 [available in the online version] for Clinician Interview Guide). Participants included medical, surgical, and radiation oncologists, dentists, dieticians, pharmacists, nurse practitioners, and oncology clinic nurses. The interviews focused on three themes: (1) the structure and function of clinic teams; (2) teamwork between clinicians and patients/caregivers; and (3) the design of an optimal surveillance and response system.

We independently interviewed eight cancer patients along with their caregivers. These interviews focused on four themes: (1) cancer care experiences; (2) people involved in care; (3) tracking of health; and (4) design of a surveillance system (see Supplementary Material S2 [available in the online version] for Patient and Caregiver Interview Guide). To ensure ongoing inclusion of the patient perspective, our research team included as co-investigators a lay cancer survivor, the spouse of a former cancer patient, and the Director of our Patient and Family Advisory Council.

Zoom interviews of approximately 1 hour in length were audio-recorded, anonymized, and professionally transcribed. Transcripts, uploaded into Dedoose, 37 were deductively coded by two qualitative researchers using a consensus-based process to detail the roles, activities, and tools/technologies involved in outpatient cancer care. 38 39 We subsequently developed role network maps of individual patient and team activities. 40 41 The contextual inquiry (i.e., observations and interviews) also informed our user interface design guidelines.

#### Iterative Design and Evaluation

Using HCD, 42 we iteratively developed prototypes of the RCS to present predicted risk scores to clinicians. Based on the design guidelines developed from clinician and patient interviews, prototypes included individual patient risk profiles and dashboards that displayed the risk profiles of all provider or clinic patients. It was critical for the RCS to be integrated into the EHR and to include key demographic and contact information for the patients. We conducted seven virtual multidisciplinary team design sessions using the MURAL platform 43 to refine the RCS prototype prior to formative usability testing with end users.

We conducted formative usability testing of the RCS with three medical oncologists, a thoracic surgeon, two nurse practitioners, and a nurse who regularly performs outpatient oncology care. To provide real-world context, we developed two realistic patient vignettes using anonymized actual patient data from phase 1. In 1-hour Zoom sessions, we presented the vignettes via the RCS prototype and asked for the clinician's feedback on the interface elements using a semistructured interview guide. At the end of each session, they completed the System Usability Scale (SUS) 44 as well as open-ended interview questions on the RCS' safety, acceptability, and ability to support workflow and patient care. All sessions were audio-recorded and transcribed. Using Dedoose, we coded each transcript for what clinicians “liked,” “disliked,” and “would like to have.” We also coded all transcripts for how the RCS could support teamwork.

## Results

### Patient Recruitment and Accrual

Seventy-one eligible patients were contacted based on our EHR-based prescreening process and clinician recommendations. Fifty patients enrolled in the study, representing a 70% yield. Of these 50 consenting patients, 5 withdrew during the study but allowed data to be kept, 4 patients died, and 41 completed the study as intended (up to 6 months). Patient demographics are shown in Table 1 .

**Table 1:** Demographics of enrolled patients ( *N* = 50)

Variable (ordered by frequency)	Value
Age	
Mean (years), standard deviation (range)	57 ± 9 (35–78)
Sex (%)	
Male Female	74% 26%
Ethnicity (%)	
White or Caucasian Black or African American American Indian or Alaska Native	86% 8% 6%
Religious affiliation (%)	
Protestant Other Catholic N/A	56% 26% 10% 8%
Education (%)	
High school/General education development (GED) Some college education Associate's/Trade School BS/BA Advanced college degree (%) N/A No diploma	24% 20% 18% 16% 12% 8% 2%
Household income (% in $1,000)	
>150 100–150 75–100 50–75 25–50 <25 N/A	22% 18% 18% 14% 12% 12% 4%
Cancer type (%)	
Head and neck Gastrointestinal Pancreatic Lung	66% 18% 10% 6%
Treatment type (%)	
Chemotherapy Radiation Surgery Immunotherapy	96% 68% 12% 6%

### Passive Surveillance Technology Use

All 50 patients provided some activity monitor data and EHR laboratory data. Over half of the enrolled participants had missing activity monitoring data due to challenges associated with the human–technology interaction, such as failure to wear the device (especially at night) or routinely charging or synchronizing the device. Even with regular reminders from the research team, these difficulties persisted. Moreover, even when the activity monitors were used appropriately, there were unexpected missing heart rate and sleep data. Seventy-four percent of patients used their smart scales routinely. Only 40% allowed access to their geolocation data.

### Active Surveillance

Patients demonstrated comfort and confidence in actively reporting PROMS (90% data capture), including symptoms, events, and CAHPS survey items. Patients submitted 347 self-reports using the MyCap app, including 305 PROMs ( Table 2 ). Additionally, a total of 229 NREs were reported through the weekly PCONES, either by patient self-reports using MyCap at home or by Research Assistant (RA)-facilitated administration of the PCONES during clinic visits. Despite these reporting behaviors, patient interviews and NRE reports revealed that ambulatory cancer patients were reluctant to “bother their doctor,” often withholding experiences of pain, severe nausea, changes in self-treatment practices (e.g., stopped taking a medication), and/or other clinically relevant events until their next scheduled clinic visit, or not divulging them entirely.

**Table 2:** Summary of patient-reported outcome measures (305 in total; *N* = 50 patients)

Measure	Value
Overall health (mean, standard deviation, 0–100 scale, low–high)	72 ± 17.6 (0–100)
Distress thermometer (mean, standard deviation, 0–10 scale, low–high)	3.6 ± 2.8 (0–10)
Care experience in last week (mean, standard deviation, 0–10 scale, low–high)	9.4 ± 1.2 (0–10)
Physical problems	
Fatigue Constipation Pain Eating Mouth sores Breathing Nose dry/congested Tingling in hands/feet Appearance Sleep Nausea Feeling swollen Diarrhea Memory/Concentration Skin dry/itchy Indigestion Changes in urination Getting around Bathing/Dressing Sexual	42% 27% 26% 25% 25% 14% 12% 11% 10% 10% 9% 9% 8% 8% 7% 6% 5% 4% 2% 2%
Emotional problems	
Worry Nervousness Fear Depression Sadness Loss of interest in usual activities	38% 19% 14% 13% 10% 6%
Practical problems	
Treatment decisions Dealing with insurance/financial issues Work/School Housing Transportation Childcare	16% 10% 5% 3% 3% 2%
Family problems	
Family health issues Dealing with children Dealing with partner	10% 5% 3%

Five clinical predictor variables were chosen from 33 candidate EHR variables based on the results of our modified Delphi methodology: patient weight, serum albumin, total protein, total bilirubin, and hemoglobin.

These predictor laboratory variables were chosen based on the potential to indicate clinical deterioration. Serum weight, albumin, and protein levels are important measures to detect protein-calorie malnutrition and cancer cachexia. Cachexia is defined as a multifactorial syndrome that results in an energy and protein imbalance with abnormal metabolism, characterized by poor appetite, loss of skeletal muscle, and weight loss. Early detection of cachexia in patients with cancer is critically important, as this can lead to poor outcomes—including poor functional status, treatment-related toxicity, quality of life, and worse survival. Moreover, to date, there are very few pharmacologic interventions that can help mitigate symptoms of cachexia and cancer-associated malnutrition, and such interventions are ineffective if cachexia is refractory and detected at later stages. This further supports the need for early identification of clinical deterioration. Similarly, total bilirubin can be used as a surrogate for hepatocellular injury, and in fact is used routinely in this manner for patients with cirrhosis (e.g., albumin and total bilirubin are used in Child–Pugh classification). Utilizing total bilirubin can help oncologists to detect earlier if there are signs of hepatotoxicity related to cancer treatment (e.g., chemotherapy, immunotherapy, tyrosine kinase inhibitors, etc.), if there is worsening liver function due to increasing tumor burden, or if there is an obstruction in the biliary system that warrants immediate attention to prevent impending ascending cholangitis or potential sepsis. Lastly, a patient's hemoglobin effectively informs clinicians whether a patient has anemia and if a blood transfusion is required. Anemia in patients with cancer can be associated with blood loss, chronic inflammation, myelosuppression from treatment, and underlying malignancy. When hemoglobin levels decline, they can manifest with physical symptoms of worsening fatigue and shortness of breath, thereby making it more challenging to administer cancer treatment safely and consistently.

Seventy-eight percent of our patients exhibited low hemoglobin and 10% had low albumin. Common symptoms reported via PCONES include Pain (89 incidents reported), Difficulty swallowing (34 incidents), Nausea (23 incidents), and Fatigue (20 incidents). Symptoms were classified into 48 different categories and each symptom was reviewed by a clinical co-investigator to determine if it was treatment-related.

Of the 229 NREs reported, 88% ( *N* = 203) occurred at home and 57% ( *N* = 131) were related to symptoms of disease. Conversely, 43% of reported NREs were related to side effects of treatment or direct treatment effects. These NREs were multifactorial with complex etiology. Only slightly more than half of all NREs were reported to clinicians or staff (often with encouragement from the RA). Table 3 shows examples of NREs.

**Table 3:** Sample of patient-reported non-routine events

**Equipment or technology-related**
Patient was having his feeding tube adjusted and the care team involved forgot to clamp the tube resulting in a leakage. Patient's advice about clamping the tube was not heeded by care team involved.
**Consequences of treatment**
Patient experienced severe nausea and cramping with chemotherapy. Began to question faith and had very dark thoughts, to the point of considering suicide. Patient waited out the nausea and pain and prayed to deal with his suicidal thoughts. Said he had sent a message to his doctors via [Confidential Patient Health Portal]. As a result, patient was waiting to see his doctors to discuss stopping chemotherapy treatments.
**Patient factors**
Patient had increasing soreness and pain over the course of a weekend, but forgot they had pain medicine that they could take. Even though this event occurred over the weekend, they did not alert their clinician about their uncontrolled pain until the following Tuesday.
Patient takes ropinirole for restless legs but forgot to take this medication before chemotherapy. Patient had an adverse response to chemotherapy involving involuntary spasms while sitting in the chair at infusion clinic. The reaction was due to the Benadryl in the infusion.

The smart scales were reliably used to capture weight during the study period. Sixty-eight percent of our patients lost weight during their enrollment period. Additionally, 18 incidents of weight loss were related to a reported NRE.

Geolocation data were not reliably captured due to frequent changes in the security protocols of Google and Google Maps, reduced patient activity outside the home during the coronavirus disease 2019 (COVID-19) pandemic, and patients' general reluctance to share geolocation data even with the research study's privacy protections in place and explained.

### Unplanned Treatment Events

Among the 50 enrolled patients, 16 patients (32%) experienced 30 UTEs (i.e., unplanned ER visits, hospitalization, or major change in care plan). Ten of these 16 patients experienced two UTEs and one patient experienced three UTEs. Contributors to these UTEs included intractable nausea and/or vomiting (six occurrences), malnourishment and/or weight loss (six occurrences), infections (six occurrences, one sepsis), hemoptysis (two occurrences), unexpected complications with their gastrostomy tube (four occurrences), chemotherapy reactions (one anaphylaxis, one angina), and one occurrence each of difficulty breathing, mucositis, severe hypokalemia, and thrombosis. Nearly two-thirds of the UTEs (63%) required hospital admission.

### Performance of Unplanned Treatment Event Risk Prediction Model

Table 4 shows the performance of four independent 7-day UTE risk prediction models using data from (1) PROMs, (2) EHR, (3) activity monitors, and (4) geolocation. The PROMs model included nine variables: Distress Thermometer, Global Health Score, NRE incidence (Yes/No), and six variables representing the occurrence of problems/concerns (see Table 2 ). The EHR model included patient weight and four laboratory variables. The activity monitor model included 10 variables: calories, sedentary minutes, moderate/high activity minutes, number of steps per day, sleep minutes, sleep episodes, resting heart rate, average heart rate, minimum heart rate, and maximum heart rate. The geolocation model included three variables: time spent at home, time spent at the hospital, and time spent at other locations.

**Table 4:** Performance metrics of independent prediction models

Model	Accuracy	Sensitivity	Specificity	PPV	NPV	F-measure	AUC-ROC
PROMs	0.989	0.74	0.99	0.84	0.99	0.900	0.98
EHR	0.989	0.77	0.99	0.80	0.99	0.625	0.98
Activity monitor	0.976	–	1	–	0.976	0.235	0.65
Geolocation	0.924	0.14	0.97	0.33	0.94	0.200	0.57

Abbreviations: AUC-ROC, area under the receiver operating characteristic curve; EHR, electronic health record; NPV, negative predictive value; PPV, positive predictive value; PROM, patient-reported outcome measure.

Several machine learning-based algorithms such as the random forest model, support vector machine, and isolation forest were trained and tested. The random forest model performed the best. The important variables identified using the feature importance algorithms were not consistent. However, the most consistent important variables were weight and global health score for active surveillance models, and maximum heart rate for passive surveillance models.

The PROMs and EHR models demonstrated moderate performance (ROC AUC 0.98). Models based on activity monitor or geolocation data had low sensitivity, PPV, and ROC AUC. The lower-than-expected performance of the prediction models can be attributed to missing and unbalanced data. Following data cleaning, approximately 42% of the collected FitBit data with all the features (relevant to the predictive model was considered in the construction of the predictive model. In the case of Geolocation data, the 2 to 10 locations designated as “healthy” and “health care” by each participant did not adequately represent their outside activity, with 86% of the participants' movements categorized as “unknown.” Due to the very high rate of missing geolocation data, the performance statistics of the geolocation model were excluded from Table 4 . Participants exhibited inconsistent PROMs reporting variables using the MyCap app, resulting in 39% missing data during the enrollment period.

The distribution of classes in the classification dataset is highly imbalanced, with minority (UTE) and majority (non-UTE) classes having proportions ranging from 2 to 3% and 97 to 98%, respectively, across various models. Despite employing the SMOTE technique to generate synthetic samples for the minority class, this data imbalance issue was not adequately resolved.

### Usability of the Risk Communication System

**Fig. 1:**
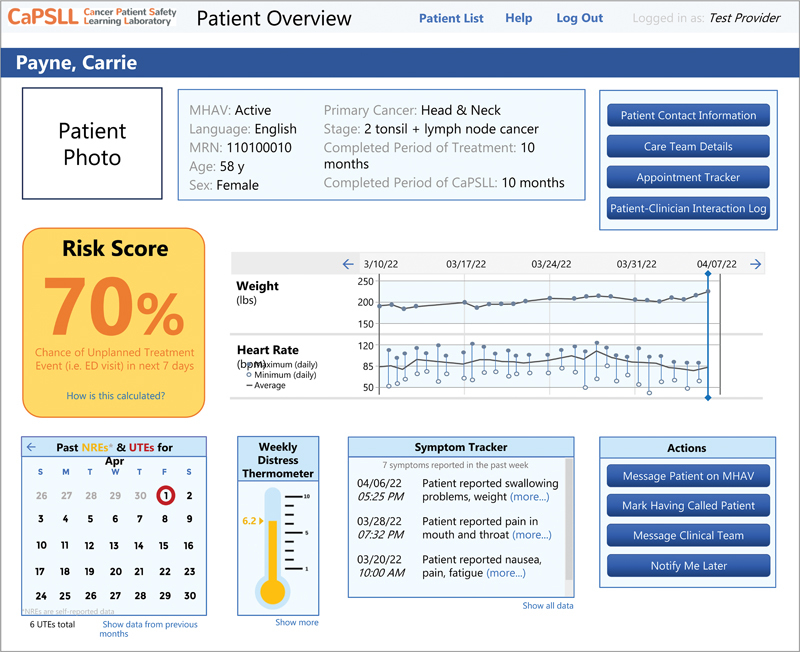
Clinician-facing RCS individual patient mock-up. Patient identity is fictitious. Data is loosely based on data observed in enrolled patients. RCS, risk communication system.

Physicians and nurses ( *N* = 7) indicated very good usability of the RCS, with an average SUS score of 76 out of 100 (median = 80; range: 60–85). 44 An SUS score of ≥72 is considered “good” (a score of >85 is considered “excellent”). 45 Clinician participants liked that the dashboard allowed them to quickly scan their patient panel and identify patients who needed attention. They also liked that the interface displayed the patient's next appointment. In the patient-specific mock-up ( Fig. 1 ), they particularly liked the trend information on patient weight. The tracker for patient symptoms (e.g., nausea, throat swelling) was also helpful.

Participants identified several areas for RCS improvement. For instance, participants recommended removing some information (e.g., patient's cancer stage, risk score percent change, time on study, activity monitor data integrity, and data from the last visit). Participants were unfamiliar with the terms “unplanned treatment event” and “non-routine event” which were replaced with a “patient event log.” Suggested additional functionality included the ability to rapidly communicate with other care team members in high-risk patients to coordinate the next steps (e.g., a phone call to the patient).

## Discussion

We describe a holistic, HCD process of creating and testing a system to surveil, predict, and communicate near-term risk for clinical deterioration of ambulatory cancer patients. While the technologies needed to build clinical surveillance systems for ambulatory care applications are commercially available and reasonably affordable, integrating these components to create a safe, reliable, and usable system that is seamlessly integrated into existing care systems remains technically and operationally challenging. Our prototype system demonstrated modest, but consequential and encouraging performance. With appreciable effort, we were able to capture 3 to 6 months of meaningful data from 50 patients undergoing ambulatory cancer treatments using low-cost commercial wrist-based monitors of heart rate and activity as well as geolocation data from smartphones, weight from an in-home Bluetooth scale, and EHR-derived clinical variables. Via a user-friendly smartphone app, we also actively captured a range of PROMs and NREs. These data were used to develop four independent predictive models of patients' 7-day risk of a UTE. The EHR and PROMs-based models demonstrated moderate predictive performance.

We also developed individual patient and clinic-wide dashboard prototypes to deliver synthesized risk information to responsible clinicians and to provide individualized information to the patients and their caregivers. Future work will be needed to implement and evaluate this promising tool for communicating risk and guiding appropriate responses.

Only a few other systems to monitor the health of cancer outpatients specifically, and complex outpatients more generally, have been reported. Two systems use electronic PROMS and activity monitors to support continuous monitoring of cancer patients in palliative care. 46 47 Owusuaa et al developed a system that uses oncology patient clinical and laboratory data, and responses to patient-directed prompts, to predict 1-year mortality. 48 Outside of oncology, Li et al 49 developed a prototype system (25 patients) to predict clinical deterioration in heart failure patients recently discharged from the hospital. This system also uses Fitbit activity monitors, cloud-based data management and processing, and machine learning to collect and analyze multimodal patient data. Our platform seems most similar in design and scope to MyPal™. 47 However, our system routinely collects weight and EHR data, and importantly, the data feed a predictive model that estimates individual risk for 7-day clinical deterioration. All these small preliminary studies, including our own, have the same goal—to improve care through improved patient engagement. However, all these endeavors have struggled to recruit and retain patients suggesting the need to better understand ambulatory patients and their relationship with technology and with the health care system.

## Limitations and Lessons Learned

The anticipated backbone of our passive surveillance system—low-cost wrist activity monitors for heart rate, physical activity, and sleep data—proved ineffective in predicting UTE risk scores due, largely, to operational barriers leading to appreciable missing data. We believe that the complexity and task burden of maintaining and wearing the activity monitors contributed to this problem. This was compounded by frequent unannounced software updates by the technology vendors which disrupted data capture or transfer. It remains to be seen whether more sophisticated and more expensive (i.e., released after the start of our study) worn monitoring technologies will overcome these barriers.

The utility of geolocation data for risk prediction modeling was limited by technical, operational, and situational factors. First, one-quarter of patients declined to allow us to track their location due to privacy concerns. Second, the study occurred during the COVID-19 pandemic when cancer patients deliberately reduced out-of-home activity. Third, extracting geolocation data required a multistep manual process to clean and reformat the data for use in the models. It is possible that with different technology and under different circumstances, geolocation data could be useful in predicting clinical deterioration.

Although this project yielded some encouraging results, an important lesson learned is that patient-centered clinical surveillance requires appreciable patient engagement and, more importantly, *patient work* . For cancer patients who already feel overburdened by their diagnosis, prognosis, and disease management activities, the perceived benefits of such a system may not justify the additional effort required. In our system, there were extensive onboarding and training requirements and the burden of daily device management. Future research is needed first to improve the reliability of the technology, to minimize the patient and caregiver burden associated with the processes and technology, and to overcome the perceived reluctance of ambulatory cancer patients to engage in patient-centered research. Then, such a system will need to be rigorously evaluated in a large multicenter trial.

## Conclusion

Despite the many challenges, we were able to use machine learning-based algorithms with PROMs, 50 51 52 53 54 55 56 57 NRE reporting, and clinical variables derived from the EHR 36 58 to predict clinical deterioration surveillance in ambulatory cancer patients. Our study provides initial evidence for designing and developing a system to monitor the well-being of cancer outpatients and predict the risk of near-term clinical deterioration. The tools and processes necessary for patient-centered surveillance-and-response systems are becoming more accessible and their functionality is rapidly improving. Simplifying device management and automating data processing will support the seamless integration and implementation of commercial wearable technologies into modern ambulatory cancer care. More work must be done to engage patients and their caregivers as members of the care team, to improve patient trust and acceptance of wearable technology, and to integrate at-home reporting of PROMs as a routine component of cancer care. We also need to remove the barriers patients currently experience or perceive in contacting their providers during times of need.

## Clinical Relevance Statement

This study demonstrates the feasibility of using systems engineering and HCD methods to design and develop a multisensory surveillance and risk prediction system for cancer outpatients. The results illustrate the opportunities and challenges of effectively implementing a surveillance-and-response system for ambulatory cancer patients.

## Multiple Choice Questions

1. In which clinical settings have continuous monitoring technologies and early warning systems been used?
  - Emergency department
  - Ambulatory clinics
  - Rehabilitation medicine
  - Inpatient hospital units
  **Answer:** The correct answer is option d. Continuous monitoring and early warning systems have been most used on hospitalized patients (inpatients). Continuous monitoring of vital signs has been implemented in inpatient settings to improve the early detection of clinical deterioration and to trigger rapid response teams.
2. Which evaluation methodology is typically used to evaluate the user experience with new technologies?
  - Randomized control trial
  - Usability testing
  - Survey
  - Focus group
  **Answer:** The correct answer is option b. Usability testing is a method of testing the functionality and satisfaction of using a web site app, or other digital product. It focuses on understanding users' experiences, thoughts, and feelings while using a product.
