# Supplementary material for: Human-Centered Design and Iterative Refinement of Tools and Methods to Implement a Surveillance and Risk Prediction System for Clinical Deterioration in Ambulatory Cancer Care
Source: ACI open. 2025 Feb 21;9(1):e18–28. doi: 10.1055/a-2437-9977 (PMC13390218; doi:10.1055/a-2437-9977)
Supplement: Supplementary file 1 — Supplementary Material [file 10-1055-a-2437-9977-s202407ra0008.pdf]

## Supplementary Material S1 Outpatient Focus Group Protocol

### Clinical Micro Teams: Clinician Version

Thank you for taking the time to talk with us today. As you may know, the goal of this project is to develop and implement a predictive model to generate a risk score to identify patients at risk for clinical deterioration in your clinic, and then implement it in the clinic. To that end, we are going to ask you a series of questions about your clinic practices. Specifically, we are interested in learning more about your care teams and how you deliver patient-centered care to your patients and their family caregivers. We hope to focus more on social, organizational, and systemic factors and routine work practices rather than individual behaviors. We will be recording today's session, but it will be transcribed in a way that your comments remain confidential. We will only report back a summary analysis of the focus groups in total after we have collected all the data.

### Theme 1: Structure and Function of Clinic Teams

1. What roles (clinicians and staff) make up your core clinic team?
   Probe: Are there roles that are not part of your core clinic team that you feel should be? If so, explain why.
   Probe: How do you introduce your clinic team to new patients (one by one, or as a group)?
2. How do you set expectations for new cancer patients and their family caregivers about their role on the team?
   Probe: After appointments/meetings, do you feel like you know what you need to look out for (i.e., signs of concern)?
   Probe: How do they contribute to your team?
   Probe: Do you feel your patients and family caregivers have a voice on your team? Are they using their voice?
   Probe: How do you ensure they share their needs, concerns, and unique circumstances?
3. What are the barriers to involving patients? When is it easier to involve them (e.g., to solve a specific problem)?
4. Describe a time when the clinical team adapted the way they normally do things for a specific patient.
   Probe: In what ways do patients and family caregivers get to offer new ways of doing things?
5. How does the clinic track and respond to clinical deterioration?
   Probe: How do you learn from this information (for patients and your team)?
   Probe: Describe the ideal system for detecting and responding to deterioration in your outpatients.
6. Thinking about the time between clinic visits, can you share a recent example of when something important fell through the cracks?
   Probe: What was the cause or source of this failure?
   Probe: What were the barriers to preventing this or recovering from it?
7. Does your clinic have team huddles?
   Probe: If "yes," what triggers a huddle (i.e., can anyone initiate one?)
   Who participates in the huddle? How is the huddle structured and what is learned during/after it?

### Theme 2: Improving Teamwork between Clinicians and Patients/Caregivers

1. What are some practices (e.g., routines, practices, etc.) you wish **your team** would do more of and/or better to improve teamwork? Patient safety?
2. What are some practices (e.g., routines, behaviors, etc.) you wish **patients and/or families** would do more of and/or better to improve teamwork? Patient safety?
   Probe: What are the opportunities to improve communication and problem-solving between patients/caregivers and their clinic team (e.g., communication of unexpected symptoms, help with medical equipment or medications, etc.)?
3. How does your clinic help patients and their caregivers avoid unplanned treatment events, for example, visits to the Emergency Department, hospital admissions, or major changes in therapy?
   Probe: How does your clinic learn about and from these events and close calls?
   Probe: How do you preserve the lessons learned?
4. What do you think the organization (e.g., Vanderbilt Ingram Cancer Center (VICC)/Vanderbilt University Medical Center (VUMC)) could change to improve teamwork (including patient engagement and communication) between clinicians and their patients and family caregivers?

### Theme 3: Design of Intervention

As we mentioned earlier, the goal of this project is to develop and implement a predictive model to generate a risk score to identify patients at risk for clinical deterioration in your clinic. Our predictive model uses data elements characterizing patients' health and physical activity (e.g., Fitbit data), activity and routines outside the home (e.g., geolocation data), clinical status (e.g., electronic health record [EHR] data), and patient-reported outcomes (e.g., symptoms, non-routine events, etc.). Based on the predictive model, we plan to alert the clinical team if the patient's risk of clinical deterioration becomes high. We would like to get your perspective on how to best implement such a risk score into your clinical workflow.

1. Who should receive the deterioration risk score information?
2. How would you like a risk score for one of your patients presented (e.g., what kind of information would you need)?
3. Probe: Would anyone like to see the risk scores of all patients (e.g., in a dashboard) or only for those at high risk of clinical deterioration? What tools/technologies should we use to deliver this information (e.g., email? My Health at Vanderbilt? Epic?)?

 © 2025. The Author(s).

4. At what point in the workflow would this information be best delivered?
   Probe: What steps would you, or others on the clinical team, need to take after receiving a risk score on a patient? How could we support these steps?
5. Are there other risk scores that you currently use in your practice?
   Probe: If "yes," what are the lessons learned from the clinic's experiences with these risk scores?
   Probe: Describe what you like and dislike about the current risk scores the clinic uses.

General Theme 3 Probes
a. How would this process be different if it occurred over the weekend/after hours?
b. What challenges do you anticipate with this project?

## Supplementary Material S2 CaPSLL Patient Interview Guide

Interview Guide: Patients and Caregivers
Thank you for being a part of our project.
[REVIEW IRB ELEMENTS AND OBTAIN INFORMED CONSENT]
[PROVIDE BACKGROUND ON THE PROJECT AND GIVE PARTICIPANT PAPER FOR DRAWING AND NOTES]

### Theme 1: Past Episodes with Cancer Care

1. How long have you been a patient at Vanderbilt Health?
   a. How long have you been getting cancer treatment?
2. Has there been a time in the last few months when something about your cancer care didn't go the way you expected? If so, could you tell me what happened?
   Probes
   - How did you (or your caregiver) notice that you were having an issue?
   - What was the first thing you did to deal with the problem? Why did you respond that way?
   - Who did you tell about this problem and in what order did you tell them?
   - When you were dealing with this problem, who did you interact with at the hospital besides your doctor(s) and nurse(s)? At home?
   - Were these people familiar to you? Were they the same people who are usually involved when you have a health-related issue?
   - In what ways was your caregiver involved when you had this unexpected health problem?
3. Can you tell me about a time when you were not able to get help when you needed it (like on the weekend)?
4. When you think of calling your care team at Vanderbilt, do you ever pause or hesitate before you call? If so, what makes you hesitate?
5. How would you describe the communication between you and your care team?
   - What leads you to describe it this way?
   - Are there any changes you would like to make to how you communicate with your care team?
   - Tell us about your comfort level with talking to someone on your care team. Who do you talk with most often? What do you usually do to reach out?
   - What causes you to reach out to your care team? Can you think of and share an example?
   - When did you hesitate to reach out to the care team? Can you tell me more about that situation?

### Theme 2: People Involved in Care

1. If you had to pick the most helpful people involved in your care, who would they be? Give us three to five people if you can.
   - Do you rely on any of these people more than others? If so, who do you rely on more and why?
   - What do these people do to help you?
   - How often do you need their help?
2. Have there been times when you were not able to do something because the right person was not with you? If so, can you give us an example?
3. Is there anything that could be done to improve your care experience at home? At the hospital?
4. Is there any kind of support you think you need that your caregivers at home and the clinical team are not giving you?

### Theme 3: Tracking Your Health

1. What motivates you to track changes in your health?
2. How do you track changes in your health? (Fitbit, the myCap app, handwritten notes, calendar, information management tool or system)
   a. How often do you use these tools?
   b. Are there any special functions or features of these tools that motivate you to track your health?
3. Do these tools or methods help you notice changes in your lifestyle (weight loss, sleep, eating)? If so, how?
4. Are there any challenges you face when you track your health with your preferred methods? If so, what are they?
5. Think about the mobile application tools you use or have used.
   a. Was there anything about the design of a mobile app that kept you from using it?
   b. Are there any features that you think you need but are missing in the tools you use?
   c. Do these apps support you in all of your health tracking needs? If not, what would you like to track that these tools currently don't track?

### Theme 4: Design

1. What kind of information do you regularly share with your providers to help them help you? Is there any other information you would like them to have?
   a. Who needs this information? (e.g., staff, doctor, nurse, etc.)

 © 2025. The Author(s).

   b. What would you want them to do with the information?
   c. How comfortable would you be with sharing this information?
2. Is there information you would like to see only after you have talked about it with your care team first?
   a. How would you feel being shown a numerical value (a number) that says something about your health at a given moment?
   b. Is this something you would want to talk about first with your provider?
3. How do prefer to learn about your health? (written-text, verbal, visual)
4. How comfortable are you filling out an online survey about your health (symptoms, non-routine events, etc.) that would be shared with your provider on a daily or weekly basis?
   a. Is there anything that might keep you from filling out this type of survey?
5. If, through the results of an online survey that you have filled out, your provider notices a health concern, how would you like them to alert you about it (email, phone, or through myHealth at Vanderbilt message)? Would you want the alert to depend on how serious the issue is?
6. How useful would you find a mobile health app that gives you suggestions about how to improve your health?
   a. What information would find the **most** useful?
   b. What information would you find the **least** useful?

Finally, are there things that can be done to improve your care experience at home? At the hospital?

 © 2025. The Author(s).
